# Evolutionary Tradeoffs between Economy and Effectiveness in Biological Homeostasis Systems

**DOI:** 10.1371/journal.pcbi.1003163

**Published:** 2013-08-08

**Authors:** Pablo Szekely, Hila Sheftel, Avi Mayo, Uri Alon

**Affiliations:** Department of Molecular Cell Biology, Weizmann Institute of Science, Rehovot, Israel; University of Chicago, United States of America

## Abstract

Biological regulatory systems face a fundamental tradeoff: they must be effective but at the same time also economical. For example, regulatory systems that are designed to repair damage must be effective in reducing damage, but economical in not making too many repair proteins because making excessive proteins carries a fitness cost to the cell, called protein burden. In order to see how biological systems compromise between the two tasks of effectiveness and economy, we applied an approach from economics and engineering called Pareto optimality. This approach allows calculating the best-compromise systems that optimally combine the two tasks. We used a simple and general model for regulation, known as integral feedback, and showed that best-compromise systems have particular combinations of biochemical parameters that control the response rate and basal level. We find that the optimal systems fall on a curve in parameter space. Due to this feature, even if one is able to measure only a small fraction of the system's parameters, one can infer the rest. We applied this approach to estimate parameters in three biological systems: response to heat shock and response to DNA damage in bacteria, and calcium homeostasis in mammals.

## Author Summary

Many systems in the cell work to keep homeostasis, or balance. For example, damage repair systems make special repair proteins to resolve damage. These systems typically have many biochemical parameters such as biochemical rate constants, and it is not clear how much of the huge parameter space is filled by actual biological systems. We examined how natural selection acts on these systems when there are two important tasks: effectiveness – rapidly repairing damage, and economy – avoiding excessive production of repair proteins. We find that this multi-task optimization situation leads to natural selection of circuits that lie on a curve in parameter space. Thus, most of parameter space is empty. Estimating only a few parameters of the circuit is enough to predict the rest. This approach allowed us to estimate parameters for bacterial heat shock and DNA repair systems, and for a mammalian hormone system responsible for calcium homeostasis.

## Introduction

Biological networks have been shown to be composed of a small set of recurring interaction patterns, called network motifs [1]–[6]. Each motif is a small circuit element that can carry out specific dynamical functions. An organism often shows hundreds or thousands of instances of each network motif, each time with different genes or proteins.

**Figure 1:**
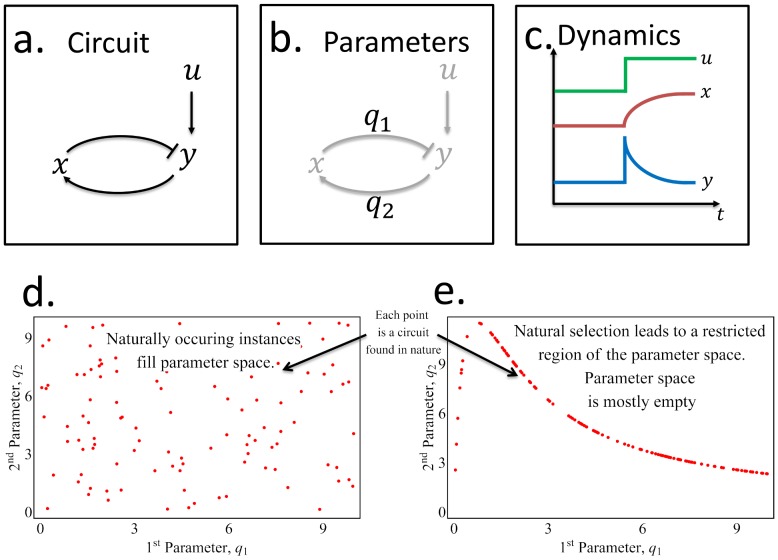
In nature, the parameters that determine the dynamics of a circuit may fill the parameter space uniformly or, instead, lie in a confined manifold within parameter space. (a) A schematic diagram of a circuit whose parameters, 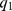 and 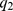 (b) determine the dynamics (c) of the internal variable (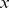, red) and the output (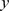, blue) for a given input time series (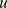, green). Two schematic illustrations of possible scenarios that could exist in nature are (d) the occurrences of the circuit fill parameter space or (e) the occurrences of the circuit are confined to a curve or manifold in parameter space. Natural selection in the context of tradeoffs can effectively remove points from (d), resulting in (e).

Qualitative aspects of the dynamics of each network motif are usually determined by their connectivity pattern - the arrows in the circuit diagram (Fig. 1a). However, in order to understand the detailed dynamics of a network motif, one needs to also know its biochemical parameters – the numbers on the arrows (Figs. 1b and 1c). If a given circuit has 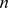 biochemical parameters, every instance of the circuit can be described by a point in an 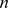-dimensional parameter space. Parameter space has been useful in theoretical studies that explore the range of dynamics accessible by a particular circuit, by sampling many points (many parameter combinations) and studying the dynamics of the resulting circuits [7]–[11]. Note that when the number of parameter is large, scanning the parameter space is a combinatorially difficult task.

An open question concerns the distribution of naturally occurring instances of a circuit in parameter space. One may imagine different scenarios: instances of the circuit may be distributed widely over parameter space (Fig. 1d), or, instead, be localized to a low-dimensional manifold within this space (Fig. 1e). The latter situation would be helpful because all the parameters of the circuit could then be derived from the estimate of only a small subset of parameters.

Recently, an analogous question has been posed for animal morphology, in which each organism is represented by a point in a space of traits [12], [13], called morphospace [14]. Animal morphology usually fills only a small part of morphospace. The range of morphology in a class of species – called the suite of variation- often falls on a line in morphospace. One theoretical explanation for such lines is that organisms need to perform different tasks, and thus face a fundamental tradeoff: No single phenotype (no point in trait space) can be optimal at all tasks. Shoval et al. [12] showed, using the concept of Pareto optimality [15]–[17], that tradeoffs often lead natural selection to phenotypes arranged on low dimensional regions in morphospace, such as lines and triangles. The vertices of these lines and triangles are phenotypes optimal at a single task, called archetypes.

Biological circuits also face multiple tasks [18]–[26]. For example they must effectively carry out a given function, but they must also economize the levels of the proteins made by the cell because unneeded proteins carry a fitness cost [27]–[32]. This tradeoff between economy and effectiveness in circuits, described by El Samad et al. [33], raises the possibility that a similar Pareto front analysis may be useful to analyze the distribution of the biochemical parameters of a circuit in parameter space.

**Figure 2:**
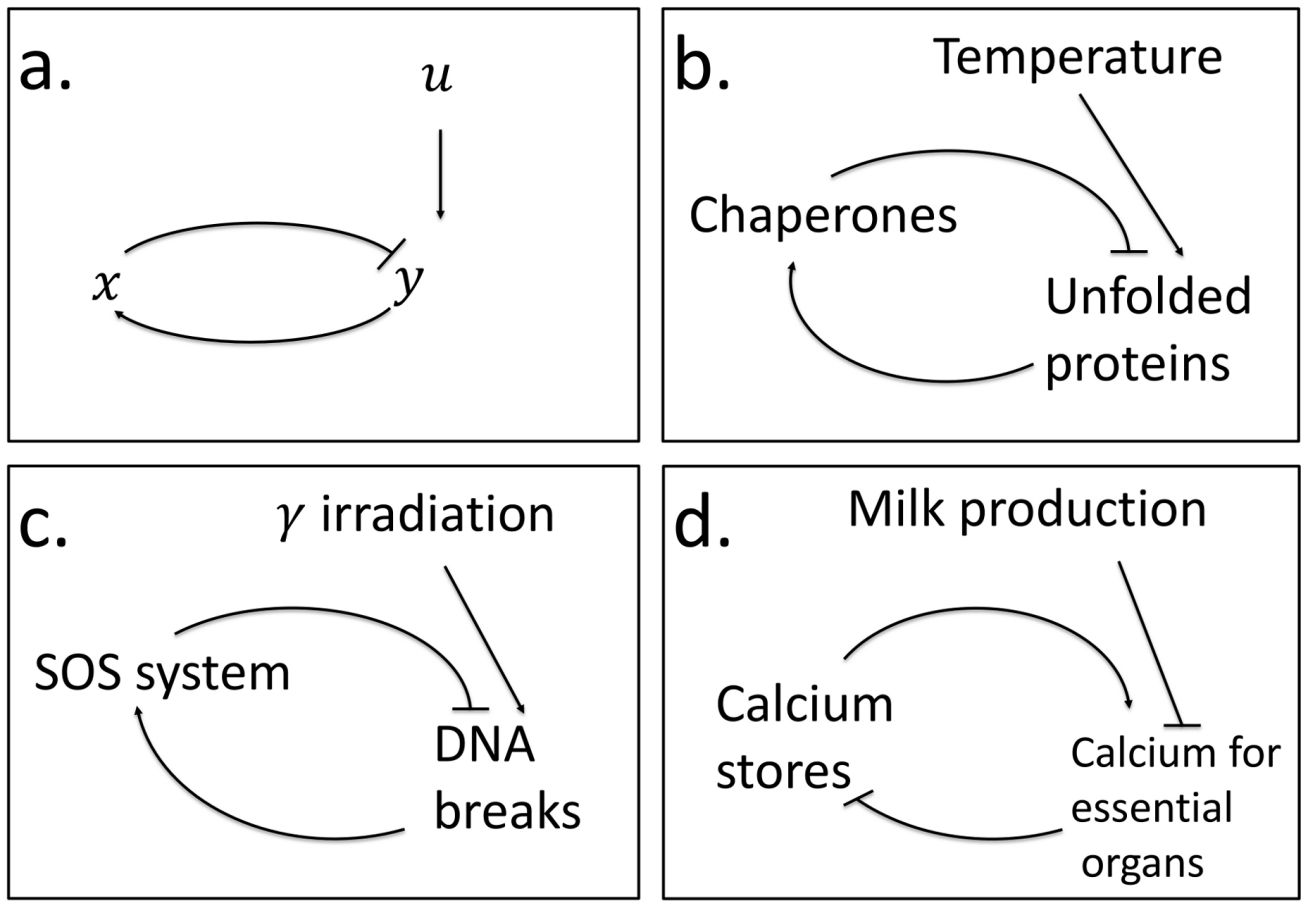
An integral feedback model for damage response and homeostasis systems. (a) An increase of the input, 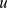, leads to a rise in the level of the output, 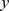, which, in turn triggers the production of the internal variable, 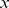, that lowers the output back to its original level. This feedback loop is at the heart of systems such as (b) the *E. coli* heat shock system - where the input is temperature, the internal variable is the amount of chaperones and the output is the level of unfolded proteins; and (c) the E coli SOS DNA repair system where the input is DNA damaging agents such as 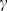 irradiation, the internal variable is DNA repair machinery and the output is the level of DNA damage. Another example is the regulation of the levels of calcium in the dairy cow (d) where the input is the calcium needed for milk production per day, the internal variable is calcium flux that goes into the blood from food, bone and other stores, and the output is flux of calcium that leaves the blood per day and is required for the activities of essential organs, such as heart and neurons.

Here, we apply such an analysis to a simple circuit, in order to exemplify an approach to study how tradeoff between tasks can lead evolved circuits to low-dimensional regions of parameter space. As a model system we study a circuit known as integral feedback- which serves as a simple model of a wide range of systems that govern physiological homeostasis, and is a mainstay of engineering feedback control. The circuit has two components (Fig. 2a): an internal variable 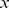 and an output 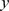. In response to an input, 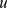, the level of 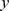 changes from its set point 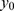. As a result, 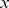 levels change slowly, causing 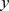 to return to 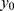. The defining property of integral feedback is that the rate of change of 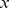 is proportional to the difference between 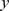 and its set-point 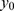, a mathematical feature that guarantees exact return to 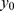 no matter what the model parameters are [34], [35].

As an example, consider the bacterial heat-shock system (Fig. 2b): unfolded proteins, 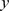, result from changes in temperature 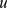. The heat shock proteins - chaperones and proteases, collectively described by 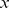, increase in level until unfolded proteins 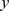 return to baseline. Integral feedback has also been proposed to describe the dynamics of DNA repair [36]–[38] and hormonal systems [35]. A detailed model of the bacterial heat shock system was previously studied by [33], suggesting that the parameters of the *E. coli* heat shock system are Pareto optimal with respect to effectiveness and economy. As in most studies that employ the Pareto front, the analysis of El Samad was in performance space. In the present study, we analyze the shape of the Pareto front in parameter space. We use a much simpler model, which has the drawback of neglecting many biological details such as non-linearity, but has the virtue of being analytically solvable and thus the shape of the Pareto front in parameter space can be solved exactly. We apply this analysis also to hormonal control and bacterial DNA repair systems. We find that natural selection with two objectives of effectiveness and economy can lead integral feedback circuits to a one-dimensional manifold in parameter space. This manifold can help to estimate difficult-to-measure parameters in each system.

## Results

### Integral feedback as a simple model for biological damage response and homeostasis systems

As a model system, we choose a well-studied class of circuits that are used to maintain homeostasis. To capture the essential behavior of these systems, we follow the models proposed by [35], [39]. These authors described the calcium and heat shock systems at various levels of detail, showing that they are essentially integral feedback loops. Here we use the simplest possible linear description of this feedback loop, ignoring important details (such as feed-forward control and non-linearity which will be treated in later sections) in order to gain clarity for analysis.

The integral feedback loop has three components. The input signal 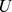 causes a change in output 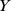 (e.g. temperature 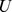 causes increase in unfolded proteins 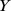). The internal variable 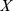 acts to reverse the effect of the input, so that 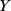 returns to its baseline level (e.g. 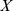 are heat shock proteins that cause unfolded proteins 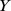 to return to a basal level). We describe these effects by a linear equation:
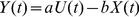
(1)


Feedback in these systems occurs because an increase in 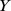 leads to production of 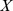, causing 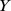 to return to its basal levels. Integral feedback is a specific form of feedback, in which the rate of production of 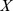 is dependent on the difference between the level of output 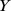 and its desired basal level 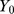:
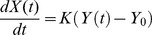
(2)


The time constant for this process is 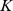. The larger 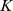, the faster 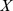 responds when 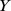 departs from its baseline 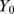. The only possible steady-state for 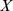 is when 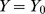. For this reason, integral feedback is a robust circuit that leads the output to its baseline, regardless of parameter values.

Note that we used the separation of timescales that occurs in the biological examples, in order to simplify the mathematical description: the production of 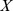 is typically much slower than the action of 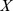 and 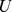 on 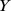. Thus, Eq 2 is a differential equation; whereas equation 1 is algebraic.

In order to reduce the number of free parameters in the model, we rescale the variables. We normalize 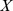 and 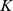 by the parameter 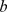 (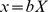,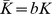), 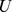 by 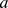 (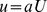). We normalize time by the typical timescale 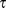 over which the system is activated, There remain only two scaled parameters, 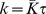 and 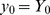. Thus we will work with the rescaled model (Fig. 2)
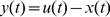
(3)

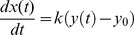
(4)


The parameter space for this model is two dimensional, with axes of 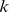 and 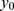, corresponding to the responsiveness rate of 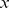 and the baseline level of 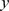. Each choice of 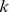 and 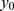 determines a particular dynamical system, which has its own characteristic dynamic response to a given change in input 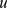. Note that the time is now measured in units of 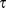.

**Figure 3:**
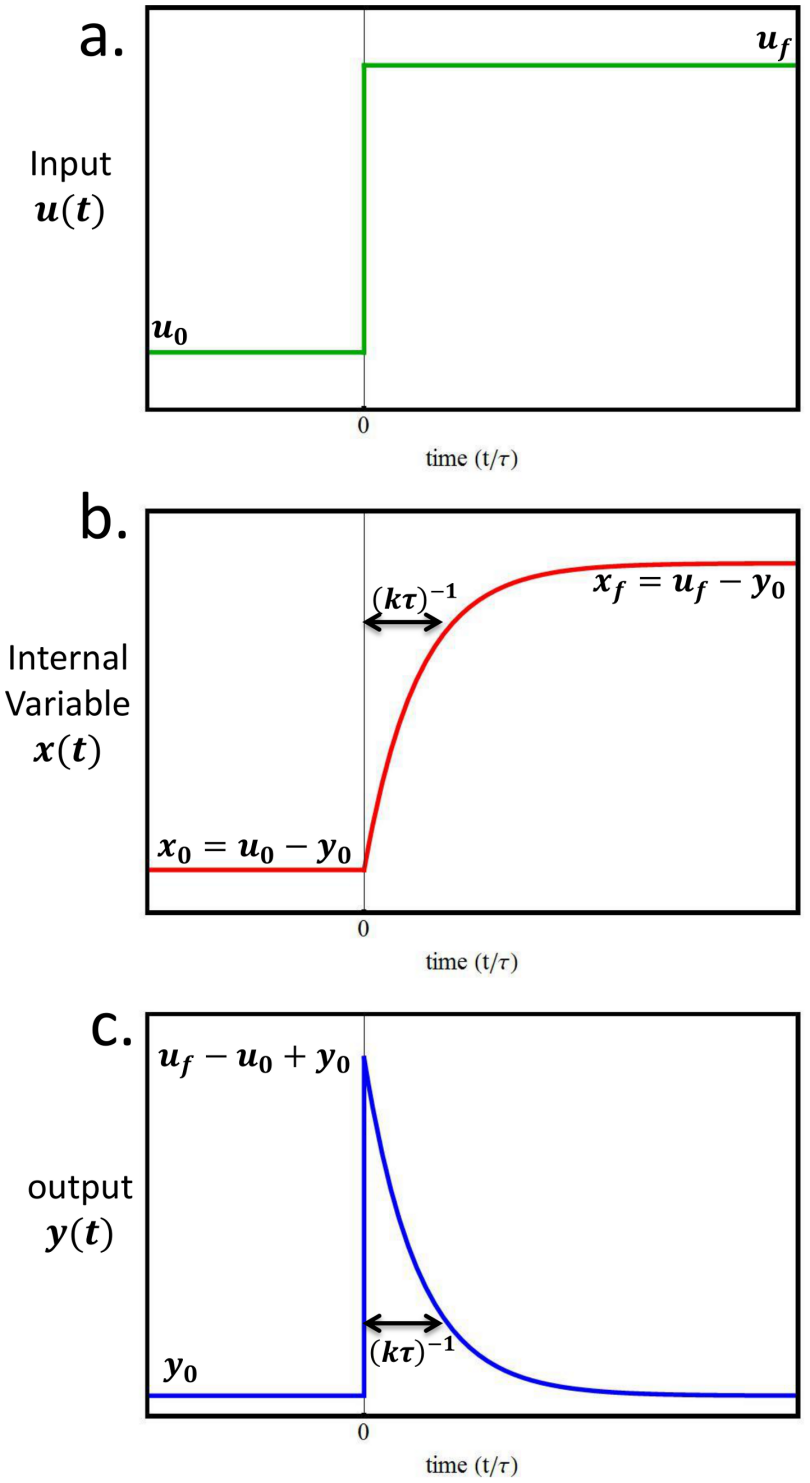
Dynamics of the integral feedback model show exact adaptation following a step change in input. (a) A step of input at 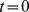 leads to (b) an increase in the internal variable level, 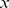. The parameter 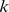 determines the rate of response. (c) The output 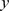 increases rapidly due to the input step, and decreases back to its baseline level 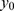 due to the action of 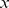.

In Fig. 3 we plot the response of the integral feedback system to a step change in input that goes from an initial level 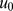 to a final level 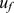, at time 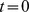. An advantage of this model is that the dynamics can be solved analytically. The internal variable 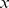 rises and exponentially approaches its new steady state
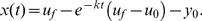
(5)


The output 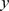 responds immediately, reaching a maximal level 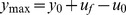. The output 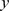 then decays due to the rise in 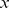, eventually returning precisely to its initial level, the baseline level 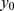


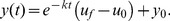
(6)


The timescale of changes in both 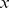 and 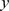 is 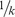.

### Tasks for an integral feedback system include economy and effectiveness

We define two tasks for the integral feedback system, following [33]. The first task is effectiveness, namely minimizing the ‘damage’ 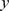. In a damage response system, the more effective the circuit, the less the average output 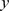, because 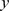 causes damage to the cell, and lower values of 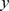 mean higher fitness. The second task is economy: the less of the proteins 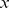 are made, the higher the fitness due to reduced protein burden [27]–[29]. There is a tradeoff inherent in these two tasks: effective systems require high levels of 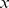, while economizing systems require low levels of 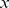. Thus, natural selection needs to compromise between effectiveness and economy.

We consider a case where the system is at steady state for a time 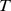, and then a step change in input occurs that lasts for time 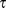 (for example, ambient temperature for time 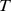, followed by temperature increase for time 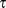). A simple choice for a performance function for the task of effectiveness, 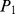, is the average output over time
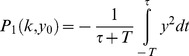
(7)And for economy, described by the performance function, 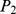 , the average of 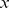 over time
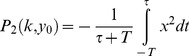
(8)


We use quadratic terms, 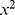 and 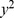, because biological cost is often an accelerating function of the cost-inducing factor [29], and because of the ease of analytical solutions [23]. Other functional forms for the performance functions lead to similar conclusions and will be discussed below.

The performance functions depend on the two circuit parameters 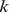 and 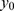: for each choice of 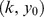, one computes the dynamics for a given step increase in input (from 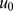 to 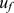), plug the dynamics 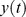 and 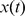 into equations 7 and 8, and computes the performances – effectiveness and economy- that characterize that point in parameter space. Analytical solutions for these equations are provided in Methods.

**Figure 4:**
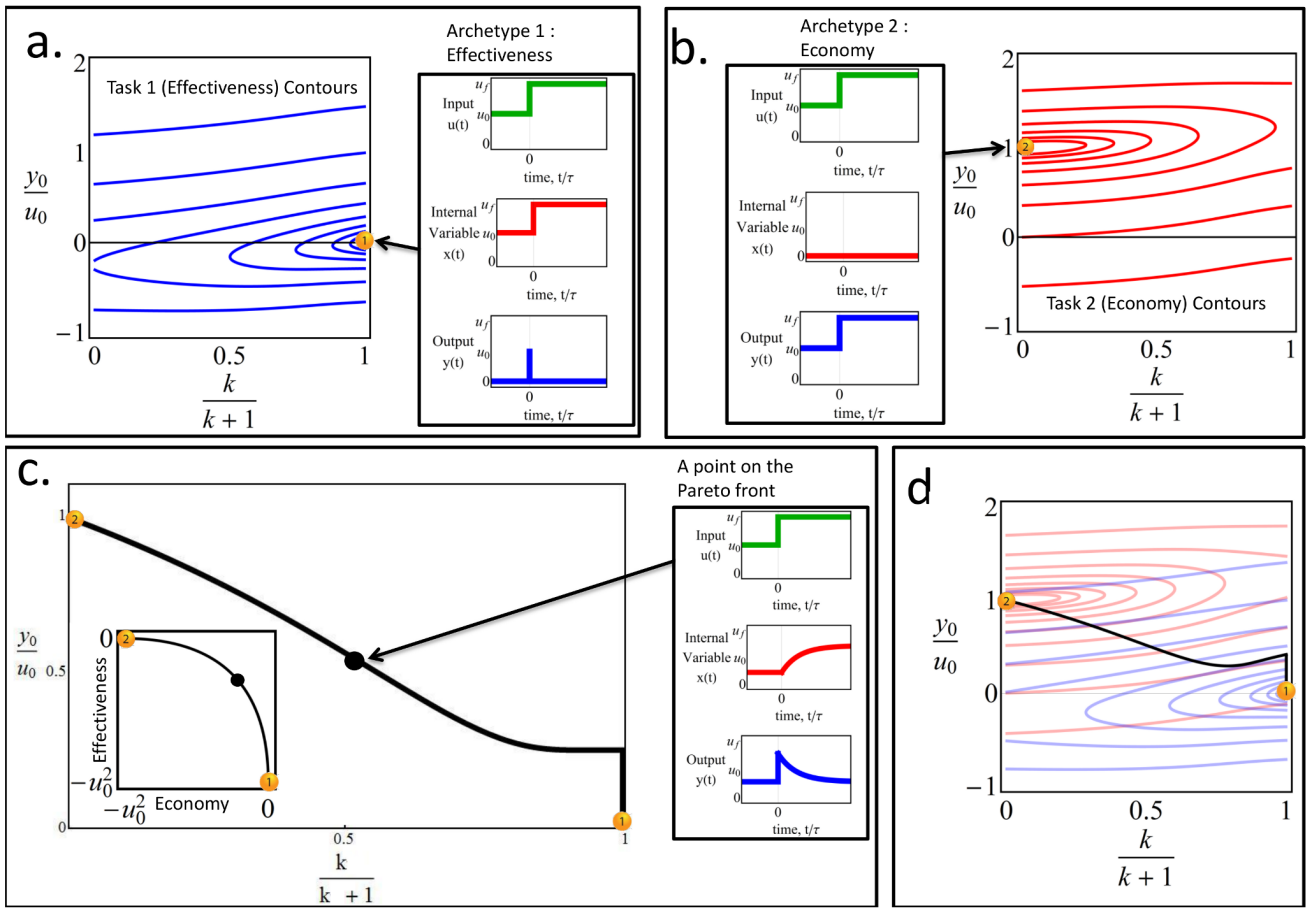
The Pareto front connects the archetypes – systems which are optimal for only one of the two tasks. The effectiveness (a) and economy (b) contours radiate outwards from the archetypes, which have their dynamics described in adjacent boxes (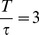). (c) The Pareto front is the set of points where the contours of the two performance functions are externally tangent. The plot shows the Pareto front when input changes are rare, that is 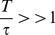. The Pareto front is a curve that connects the two archetypes. In the inset the Pareto front in performance space- note that axes are the effectiveness and economy, not the biochemical parameters as in parameter space of (a)–(c). The archetypes have the maximal performance in their respective tasks. An analytical solution shows the front is a parabola in performance space (see Methods). (d) the overlay of the contours of (a) and (b), and the resulting Pareto front (See Fig. 6 for further details).

In Fig. 4a, we plot the contours of effectiveness in parameter space- lines of equal 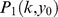 Parameter space is plotted with 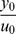 on one axis, and 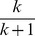 on the other axis. The latter is a way to present an infinite range of 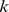 in a compact way (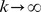 means 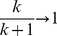).

Effectiveness (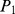) is maximized at a point that can be called the effectiveness archetype, at 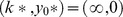. This archetype system is an extreme (limiting) case in which economy does not factor at all into consideration. It has an infinitely brief rise in 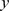 after a step change in the input, caused by an infinitely rapid increase in 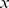. This archetype effectively makes an infinite amount of 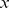 in order to speed the return of 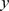 to the baseline. Contours of performance at task 1 radiate around the archetype in elongated rings (Fig. 4a).

Economy (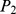) is maximized at a different point, the economy archetype (archetype 2), at 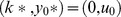 . This too is an extreme case where effectiveness is not a consideration. Here no 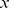 is produced at all (so that economy is maximal). As a result, 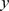 responds in an unmitigated way to the change in input, without returning to baseline. In effect, this archetype is akin to a loss of the response system 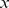. Contours of decreasing economy (increasing 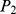) surround the archetype in elongated rings (Fig. 4b).

### The Pareto front is a curve in parameter space that best compromises between the tasks

We next computed the Pareto front [12], [20], [40]–[42], defined as follows. We term point 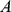 in parameter space as dominated by point 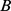 if the performance in both task 1 and 2 is better at 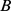 than in 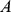 (that is 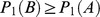 and 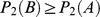). Because biological fitness is an increasing function of 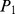 and of 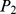, point 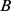 has higher fitness than point 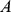. As a result, natural selection would tend to select against systems at point 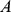, and they would vanish from the population. The Pareto front is the set of points that remains after all points dominated by another point are removed. The Pareto front thus represents the maximal set of phenotypes that will be found given that natural selection is the main force at play.

The Pareto front is a powerful concept because it does not require knowledge of the precise shape of the fitness function, as long as fitness is an increasing function of both performances. The exact shape of the fitness function, 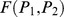 determines which point along the front is selected.

We calculated the Pareto front in parameter space [20], [43], [44] (Fig. 4d). For this purpose, we used the fact that the Pareto front is the locus of points at which the contours of the two performance functions are externally tangent [12], [13]. This allowed an analytical solution of the front (see Methods). We tested the analytical solution by numerical simulations in which points dominated by other points were removed in evolutionary simulations (see Methods).

We find that the Pareto front, in the case where input changes are rare 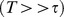, is a curve that connects the two archetypes (Fig. 4c). Its formula is 
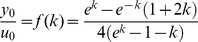
. Thus, most of parameter space is predicted by this theory to be empty, and natural systems are expected to fall on a curve. Interestingly, the front does not depend on the final input value in the step, 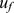, but only on the ambient input 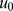 (See Methods). When effectiveness is more impactful for fitness than economy 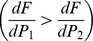, systems should lie on the front closer to the effectiveness archetype - with lower baseline 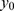 and faster responsiveness 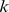. When economy is more impactful for fitness, systems should lie on the front closer to economy archetype, with higher baseline 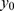, and slower responsiveness (lower 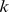).

**Figure 5:**
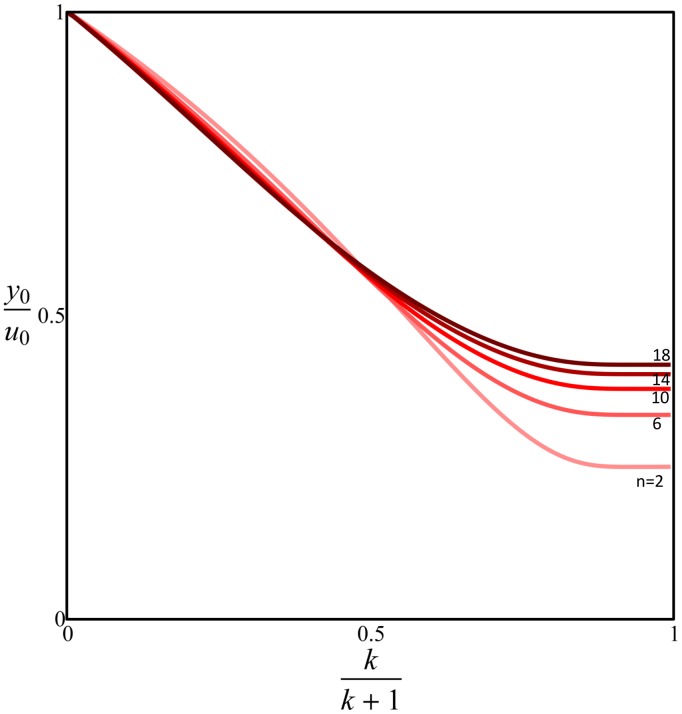
Altering the mathematical description of the performance functions does not cause substantial difference in the Pareto front shape. We changed the integrand power in both tasks from 2 to 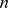 (equations 7 and 8). The calculated front uses 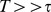 (method).

We tested the sensitivity of the Pareto front to variations in the form of the performance functions (Fig. 5). We tested
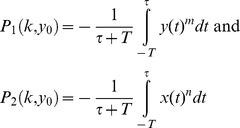



We find that changing the powers 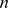 and 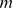 between 1 and 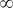 had only minor effects on the shape of the front. The higher 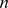 or 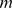, the higher the baseline value 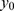 of the effectiveness end of the front. The front is insensitive to performance function shape at the economy end. We also tested other functional forms of the performance functions and find similar insensitivity of the front shape (Fig. S1 and S2).

We also tested the sensitivity of this analysis to changes in the integral-feedback model itself. We added feed-forward control, known to occur in the bacterial heat shock system, by changing the parameter 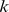 into 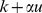, allowing the input 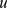 to directly affect the internal variable, 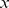 . This describes the effect of input signal on the responsiveness of 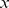. Since we consider step changes in 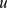, the present analysis applies precisely to this case as well, when one adjusts 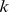 by the value of 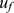. The resulting Pareto front is identical to Fig. 5, with appropriate change of 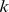 to 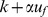. We also tested the model by adding non-linearity to the equations, and by removing the assumption of separation of timescales between 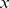 and 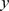. The results are detailed in Fig. S3, and generally show that the qualitative conclusions of a Pareto front curve, which connects the two archetypes and is insensitive to the form of the performance functions, remain valid.

**Figure 6:**
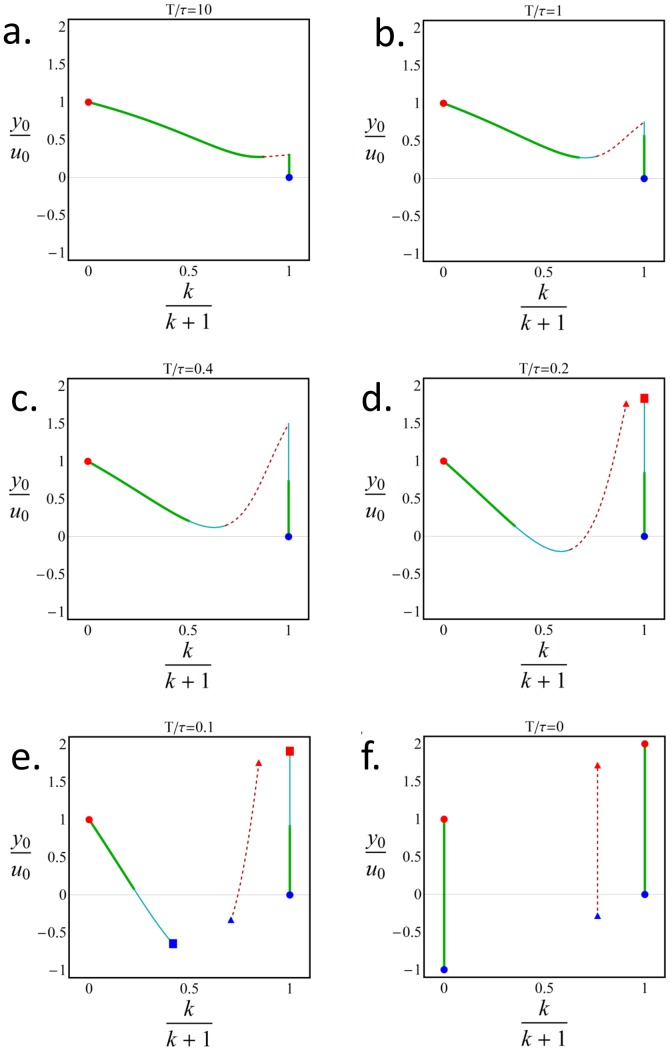
When input changes become frequent, the Pareto front shows complex changes in shape. We plot the Pareto front changing the parameter 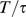, which describes the typical duration between input changes. The archetypes of effectiveness and economy (marked in blue and red, respectively) are connected by the Pareto front (green) , which for any finite 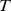 is split into two parts. For 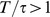 the Pareto front resembles its limit of rare input changes (a–c). As 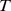 gets smaller a local Pareto front (cyan) emerges and a separatrix emerges (red-dashed) and grows. When 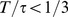 (d) a local minimum (square) and a saddle point (triangle) emerge for the economy task. And when 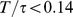 (e) the same occurs to the effectiveness task and the branches of the Pareto front becomes disjoint, until 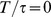 (f) where two parallel lines emerge. Red dashed lines are points where contours are tangent but are not part of the Pareto front (see methods).

It is likely that many damage response systems evolve in the limit when input changes are relatively rare 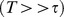. For completeness, we also studied the Pareto front when changes in input are more frequent (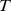 comparable to 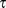) (Fig. 6) [45]. In this case, unexpected complexity was found in this simple model system. As long as 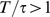, the front is a curve resembling Fig. 5 that connects the two archetypes, with an unstable region near archetype 1, at which the front jumps to 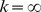 (Fig. 6a,b). At 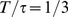 the front splits into two disjoint components, one of which is a range of 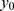 values with 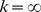 (Fig. 6d). At 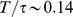, the front splits again into two disjoint curves separated by an unstable ridge.(Fig. 6e,f).

### Heat shock, DNA repair and calcium hormone system parameters may be inferred from the Pareto front

#### Heat shock system

**Figure 7:**
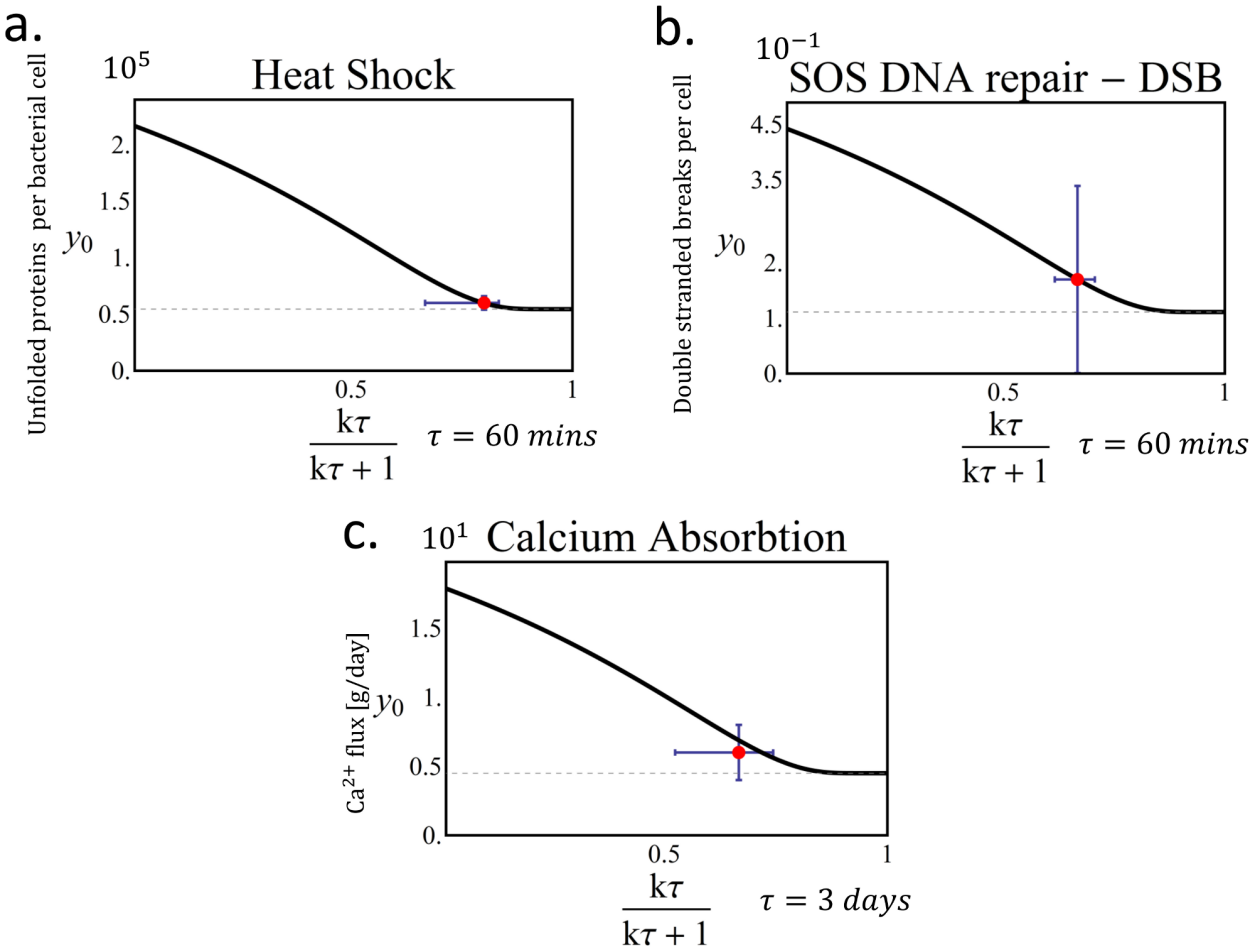
Parameters for three biological systems can be estimated from the Pareto front. Three examples of biological systems that can be modeled by an integral feedback circuit agree with the model's prediction. In all, the curve is the Pareto front in the case of a rare input change; the point represents the specific values for each example. (a) in the heat Shock system (b) the DNA damage repair system of *E. coli*, and (c) in the regulation system of the calcium in dairy cows. Values for heat shock and calcium systems were estimated independently and showed good agreement with the theory. In the SOS DNA repair system (b) we fitted using the model the value of the basal input (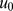) by knowing the time scale (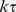) and set-point (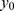) of the system, which provides an estimate of the basal level of DNA damage in the absence of a DNA repair system. In all the data sets the error bars represent different estimates of the values.

Finally, we explore the implications of the Pareto analysis for three biological examples of homeostasis systems (Table 1). We begin with the heat-shock system of *E. coli*. The baseline level of unfolded proteins at ambient temperature (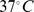) has been estimated to be about 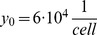 [39], which amounts to about 2–3% of the total protein in a growing cell. The responsiveness parameter of the system, 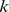, can be estimated from the typical timescale at which unfolded proteins are removed by the heat-shock system, which is about 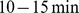 [39]. Considering the dynamics over a cell generation time, so that 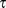 = 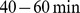, yields 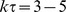. Plotting this point on the Pareto front (Fig. 7a) suggests that it lies towards the effectiveness archetype, in a relatively flat region of the front at which the value of 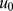 can be robustly estimated as 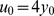. This suggests a value of 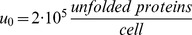 . This value can be interpreted to mean that without a heat-shock system, at ambient temperature, *E. coli* would have had 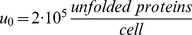, amounting to about 10% of its total protein. This level agrees with the estimated lethal limit of unfolded protein [46], and with the fraction of proteins that require extra chaperone assistance to fold as they exit the ribosome [47].

**Table 1:** Summary of the data used to compare to the Pareto front in Figure 7.

	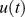 – input	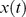 – internal variable	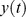 - output	Units of 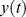 , 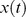 and 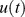	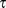	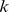	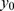	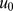
The heat shock system of *E. coli*	Temperature – The amount of unfolded proteins the system would have in the hypothetical case of having no heat shock system	Chaperones – The average amount of unfolded proteins that each unit of cell machinery folds in its lifetime	Unfolded proteins	Unfolded proteins	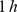 typical generation time	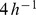 Inferred [39]	60,000 [39]	200,000 [47]
Calcium blood concentration in dairy cows	Flux of calcium for milk production	Flux of calcium to the cow from bone and intestine	Flux of calcium for vital organs	g/day	3 days [54]	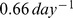 [35]	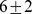 [55]	18 [54]
DNA SOS Repair system	Amount of DNA damage inflicted	Amount of DNA damage fixed by the system	The amount of DNA damage remaining	Double stranded breaks	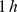 typical generation time	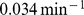 [38], [49]	0.17 [50]	0.44 - Fitted

In this example, the Pareto front allows estimation of the amount of unfolded protein expected without a heat-shock system, a value that is otherwise difficult to study because knockout of the heat shock system is lethal at ambient temperature [48].

#### DNA repair system

The second example is DNA damage repair in *E coli*. Here the timescale for the response to 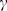 irradiation, which causes double stranded DNA breaks (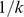), is about 20 min [49] (similar to timescale for response to UV damage [38]). By taking 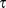 as the cell generation time, 40–60 min, we find that 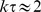. The baseline level of double stranded DNA breaks is 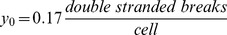 [50]. Using the Pareto front (Fig. 7b), one can estimate the level of damage expected if there was no repair system and no irradiation, 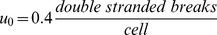 .

Note that detailed experiments and models of the SOS repair system and its mammalian counterpart show additional features such as multiple pulses of repair enzyme production [38], [51], [52]. These features are not accounted for in the present model. Future studies may include mutagenic repair as an additional potential task, perhaps with a new performance function 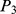 [53].

#### Calcium homeostasis hormonal system

The final example is control of calcium blood levels in mammals (see Methods for model). Data from dairy cows shows that after giving birth, calcium levels drop primarily due to milk production. In response, the hormones PTH and 1, 25-DHCC rise, leading to release of calcium from body stores. Calcium blood levels return to baseline exponentially with a time constant of about 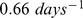 [35]. In some cases, failure to recover baseline calcium levels leads to sickness (parturient paresis), which can be prevented by injecting calcium. As an estimate of 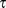 we use 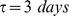, the time until an untreated cow typically shows signs of sickness [54], resulting in 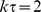. From the Pareto front, this yields 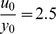 (Fig. 7C).

We can compare these values to an independent estimate. We interpret 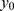 as the amount of calcium needed per day by a cow, estimated to be 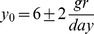 [55]. The extra loss of calcium due to milk production, which we interpret as 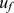, is about 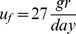 (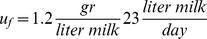, [56], [57]). The treatment for a cow with parturient paresis, which is caused by a failure to restore calcium levels following parturition, is to inject 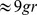 of calcium to its blood stream [54]. Hence, 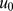 is estimated to be approximately 
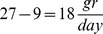
. This yields 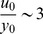, in reasonable agreement with the value from the Pareto front, 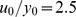.

## Discussion

This study examined how natural selection acts on a simple biological circuit when two tasks are important: effectiveness and economy. We find that this multi-task optimization situation leads to natural selection of circuits that lie on a curve in parameter space. Thus, most of parameter space is empty. The curve is the Pareto front, composed of best-tradeoff circuits, and connects two archetype points in parameter space. These archetypes represent circuits optimized for only one of the two tasks. The simple model of the integral feedback circuit enabled analytical solution of the front shape.

The resulting Pareto front allows estimation of parameters in several example systems, bacterial heat shock and DNA repair, and mammalian calcium homeostasis. Interestingly, all three examples are in a plateau region of the Pareto front, in the side tending towards effectiveness. This may result from diminishing returns [58], in which speeding up system response (increasing 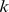) leads to small increase in effectiveness but a large increase in protein cost. In this plateau, a simple relation is found between the basal input and basal output, 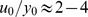. This means that large gains (large suppression of damage 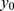 by the basal activity of the system), are not possible, at least in this simple model. Large gains can only be reached at very large 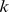, which may be unfeasible in terms of cost.

Points located in other regions of the Pareto front curve are expected in organisms which have different relative fitness contributions from the two tasks. For example, *Buchnera*, a relative of *E coli* which is an obligate symbiont of termites, has a heat shock system, but its proteins do not seem to change their expression level upon heat stress [59]. In this system, economy may outweigh effectiveness, due to the rarity of heat stress in the environment in which *Buchnera* evolved; accordingly, a solution close to the economy archetype seems to have been selected. Throughout the *Buchnera* genome, evidence of economy is prevalent [60]–[63].

Previous studies of Pareto optimality of biological circuits [12], [13], [20], [21], [64], engineered circuits [65]–[67], and of metabolic fluxes [64], [68] have usually focused on performance space. El Samad et al. [33] found that the *E coli* heat shock system is on the Pareto front in performance space, and other studies compared different circuits theoretically in terms of hypothetical tasks in performance space [20], [21], [25]. Lan et al [24] presented a statistical-mechanics based analysis of the tradeoff in the bacterial chemotaxis between energetic cost and adaptation error. Recently, Barton and Sontag [25] analyzed the tradeoff between insulation and energetic cost of signaling . The present study computes the shape of the Pareto front of a biological circuit in *parameter space*, rather than performance space. This leads to the possibility of estimating difficult to measure parameters. The present study aims at categorizing best-tradeoff instances of the same circuit, rather than comparing between different circuit topologies [11], [18], [19].

Other optimization methods are also helpful in understanding tradeoffs. Variational calculus was employed to optimize temporal profiles of enzymes with respect to cost [23]. Optimal control using Pontryagin's method was applied to understand the optimal dynamics of wasp reproductive strategies [69], and the order of developmental events in the mouse intestinal crypt [70].

The conclusions of the present study can, in principle, be tested experimentally. Doing so requires measuring the parameters of a circuit accurately, a difficult task which is becoming more feasible with advances in experimental technology. It would be instructive to attempt a comparative analysis of the parameters of a given circuit in different organisms. For example, measuring 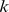 and 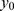 in heat shock systems or DNA repair systems in different bacterial species can test whether these systems all fall on a curve in parameters space. The position of each organism on this curve should correspond to the relative importance of effectiveness and economy in its natural environment. The present theory would be contradicted if the points fill most of parameter space, instead of falling on a curve or other low dimensional manifold.

One such empirically discovered curve was found in the analysis of the biochemical parameters of Rubisco, an important carbon fixation enzyme. The enzyme affinities and velocity parameters from different plants and microorganisms fall on a line in a four-dimensional parameter space [71] . This may represent tradeoffs between efficiency and specificity of the enzyme.

The present study analyzed the case of two tasks. When there are a larger number of tasks, theory [12] suggests that Pareto fronts should resemble polygons whose vertices are the archetypes: points in parameter space that optimize a single task. Thus, three tasks should lead to fronts that resemble a full triangle; four tasks should lead to a tetrahedron etc. If only a single task exists, natural circuits should all fall on the same point in parameter space, the point that maximizes the task performance (and therefore maximizes fitness). Analyzing multi-task cases for biological circuits is an interesting avenue for further research. Analyzing the dynamical behavior of other common network motifs in terms of multiple tasks, such as feedforward loops [3], [4] and autoregulation [72]–[74] would be fascinating as well.

## Methods

### Analytical solution for performance functions

In order to find the Pareto front we first calculate the performance functions of effectiveness and economy normalizing 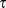 to be 1:
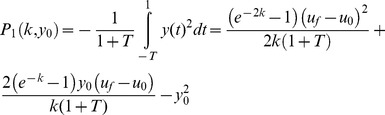
(9)

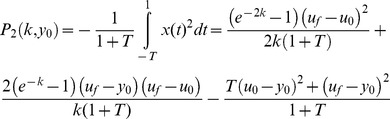
(10)


The relation between both performance functions is parabolic, and given by:
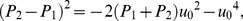
(11)We searched in each of the performance functions for extremal points, by solving for when the derivative of performance functions according to 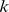 and 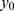 equal 0. Each point was then classified either as a maximum or a saddle point depending on the value of the determinant of the Hessian (matrix fo second derivatives). The set of equations was solved numerically. For each performance function, we find a critical value of 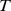, called 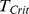, at which behavior changes qualitatively (Fig. 6). When 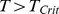 one maximum point is found, and when 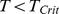 two maxima and one saddle point are found.

### Analytical solution for the Pareto front

The Pareto front is the locus of points at which contours of the two performance functions are externally tangent, namely points 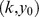 at which
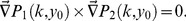
(12)


In the two dimensional case this is equivalent to solving
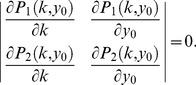
(13)


Externally tangent points must further fulfill
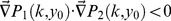
(14)


More tests are needed in case where the tangent contours intersect at points away from the tangency point (this does not occur for the case 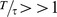).

We isolate 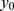 to obtain an expression for the Pareto front:
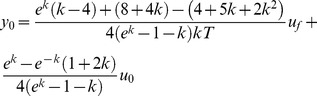
(15)


The solution corresponds to externally-tangent contours only in the region confined between the following contours in parameter space:
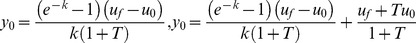
(16)


When input changes are rare, 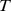 is large (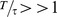), and the limiting Pareto front is
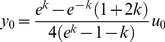
(17)and is confined to the region between 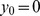 and 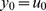. Equation 15 is confined entirely in this region.

Please note that the contours of the performance in the limiting case of 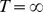 are all parallel to the 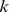-axes and to each other, and thus their tangency points cannot be calculated. To calculate the Pareto front in this limit, we calculated the front at finite 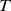 and then took the limit 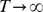.

If the equal-performance contours of the performance functions are convex, the tangency point between them is on the Pareto front. However, in the case of finite 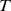, some of the contours are not convex. This results in a region where contours intersect each other and change their curvature before touching each other. Hence, the resulting tangency points, that when looking locally seem like external tangency points, are actually internal tangency points. Such tangency point are dominated by other points in trait space and are not Pareto optimal. This leads to a situation where the above region that lies on the curve connecting the archetypes is not Pareto optimal. We denoted such points by red dashed lines in Fig. 6. [13].

Another section marked in Fig. 6 in cyan describes points that lie on externally tangent contours, but the contours intersect each other in a different region of the parameter space, resulting in a dominance of points in the intersection region between the contours over the tangency points. Such points are said to be “locally Pareto optimal”, and the region were they lie is termed “local Pareto front”..

In order to test our analytic results, we performed simulations on a population of points evenly distributed in the parameter space [75]–[78]. For each point we calculated the two performances, and eliminated all the points that were dominated (outperformed in both tasks) by another point. We added some noise to the remaining points and repeated the comparison; we repeated this cycle several times. This helped us to overcome the effects of finite number of sampling points. The simulations were in excellent accordance with the analytical results. (Fig. S4).

### Model for calcium system

In the calcium system, the dynamics are somewhat different than in the heat shock system. The sign of 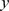 (level of calcium in blood) is negative, because when 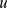 rises (calcium demand) 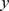 gets smaller and 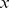 (calcium flux into the blood cycle) restore the level of 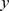 back to normal, resulting in the following model:
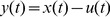
(18)

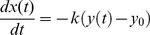
(19)


The Pareto front for this model is identical to that of the model above.

## Supporting Information

Figure S1 **The basic monotonic shape of the Pareto front is robust to the value of the integrands' power of the two tasks.** The gray line represent the original {n,m} = {2,2} tasks used throughout the paper, the label above each graph represent the power of the integrand of the economy and effectiveness tasks n and m, respectively,
(TIF)

Figure S2
**When taking both integrands' powers together toward infinity, the Pareto front converges.** The Pareto front for any n = m always begins from 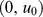 and reaches the value in the graph as 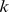 goes to infinity.(TIF)

Figure S3
**The Pareto front for a case of nonlinear integral feedback with no separation of time scales.** We extend the model in the main text by adding a time dependent ODE for 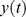. In natural systems, the approximation that 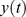 is much faster than 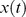 is reasonable. We also added nonlinearity in which 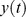 decay is multiplicative in 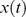, at rate 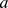. This is a reasonable model of damage repair systems in which the repair proteins 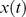 interact by mass action kinetics with the damage 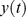. This results in 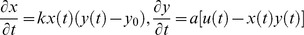. Performance contours are in red and blue. Black lines are lines where performance contours are externally tangent. Green dots are the Pareto front according to simulations (see Fig. 4s for details). The qualitative conclusions of the main text remain valid: Pareto front is a curve that connects the economy and efficiency archetypes.(TIF)

Figure S4
**Simulations concur with the analytical results. Simulated data falls on the stable branches of the analytical solution for the Pareto front.** Here, 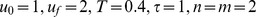 . Simulation used an initial population of 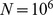 randomly and uniformly distributed points. Points dominated in both tasks by other points were removed. Surviving points were perturbed by small noise (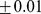), and the process was repeated for 60 iterations, reducing the amplitude of the noise gradually to (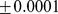). For comparison to Pareto simulation approaches see [75]–[78].(TIF)
