# Supplementary figures and images for: Evolutionary Tradeoffs between Economy and Effectiveness in Biological Homeostasis Systems
Source: PLoS Comput Biol. 2013 Aug 8;9(8):e1003163. doi: 10.1371/journal.pcbi.1003163 (PMC3738462; doi:10.1371/journal.pcbi.1003163)

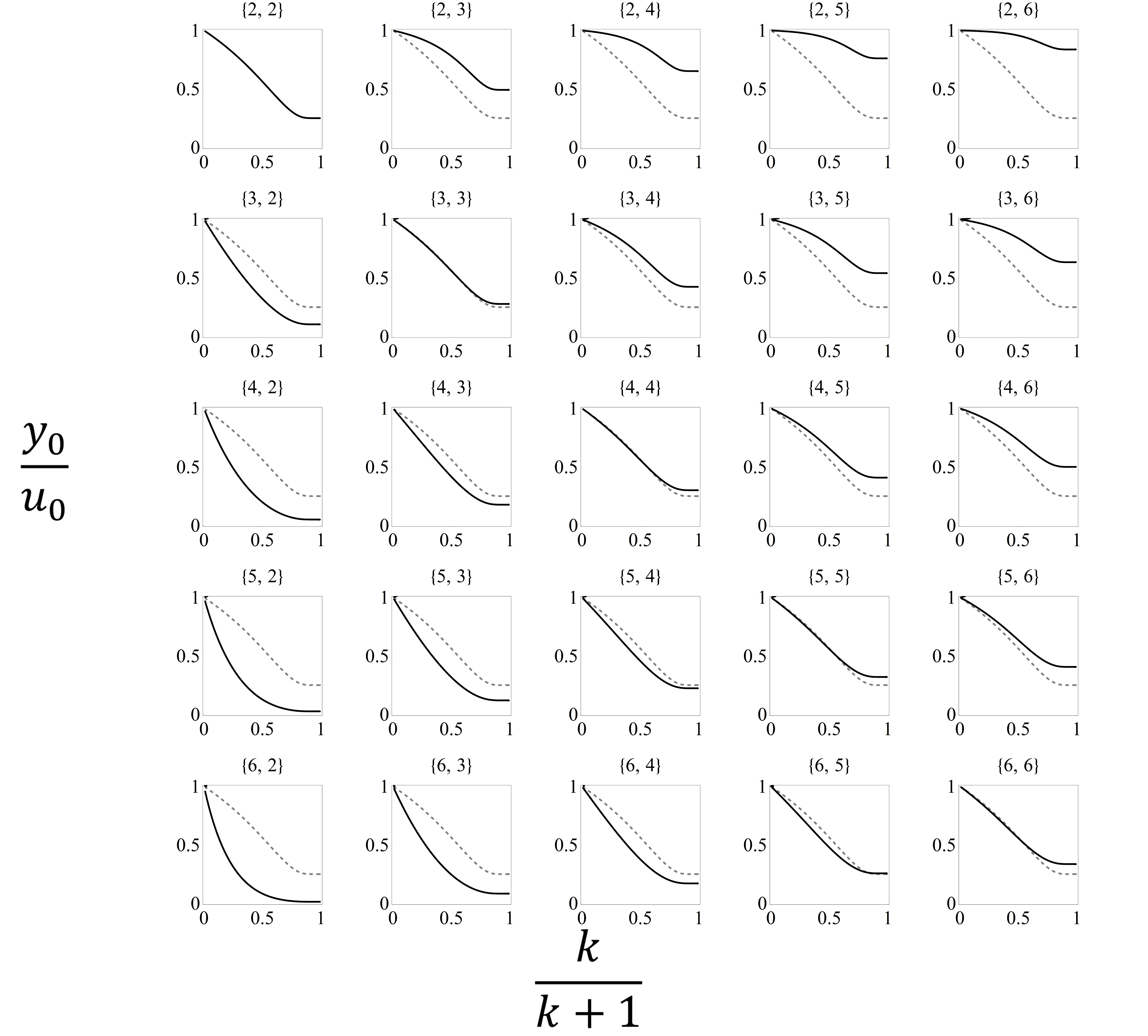

Supplement: Figure S1 — The basic monotonic shape of the Pareto front is robust to the value of the integrands' power of the two tasks. The gray line represent the original {n,m} = {2,2} tasks used throughout the paper, the label above each graph represent the power of the integrand of the economy and effectiveness tasks n and m, respectively, (TIF) [file pcbi.1003163.s001.tif]

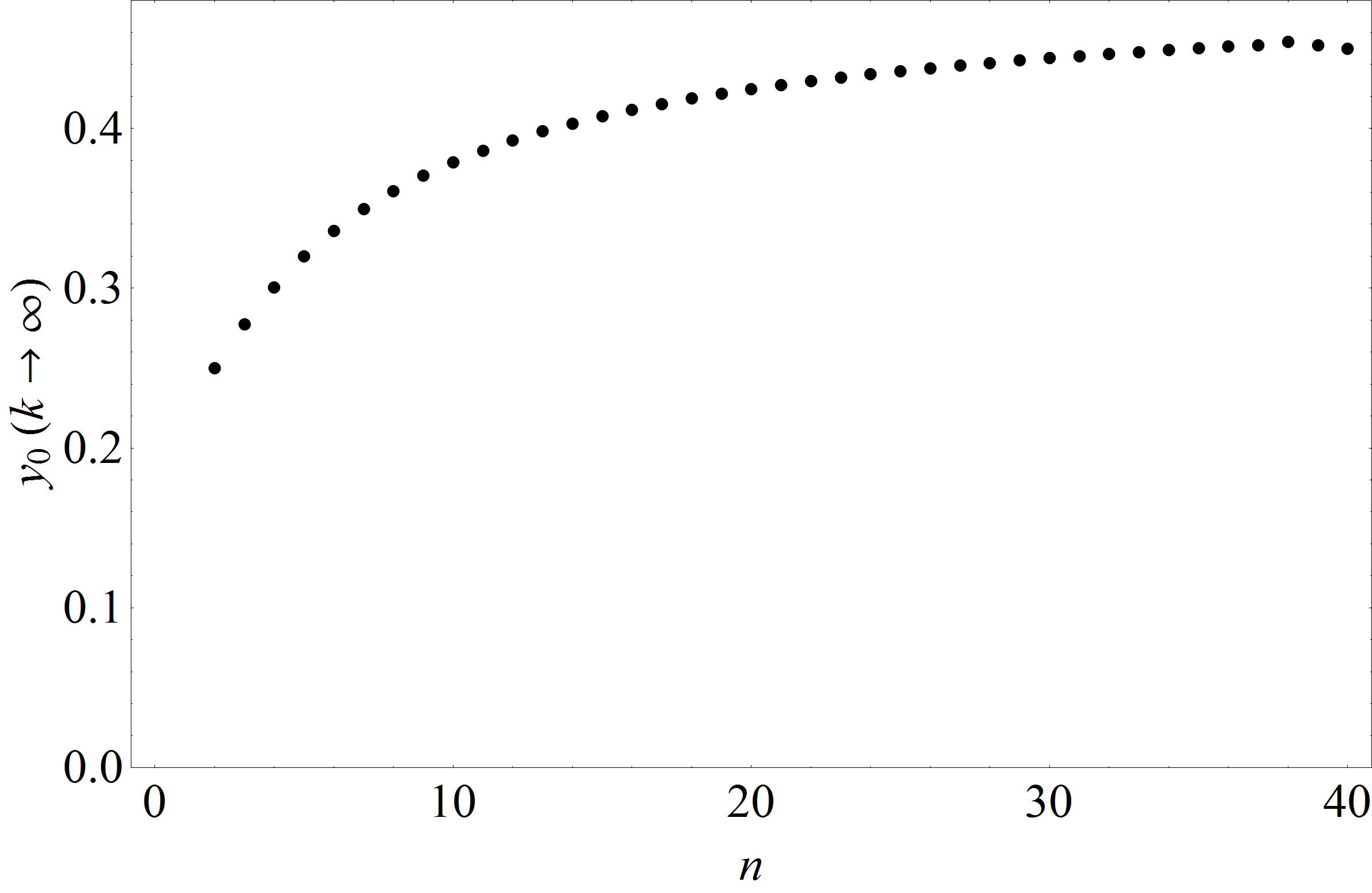

Supplement: Figure S2 — When taking both integrands' powers together toward infinity, the Pareto front converges. The Pareto front for any n = m always begins from and reaches the value in the graph as goes to infinity. (TIF) [file pcbi.1003163.s002.tif]

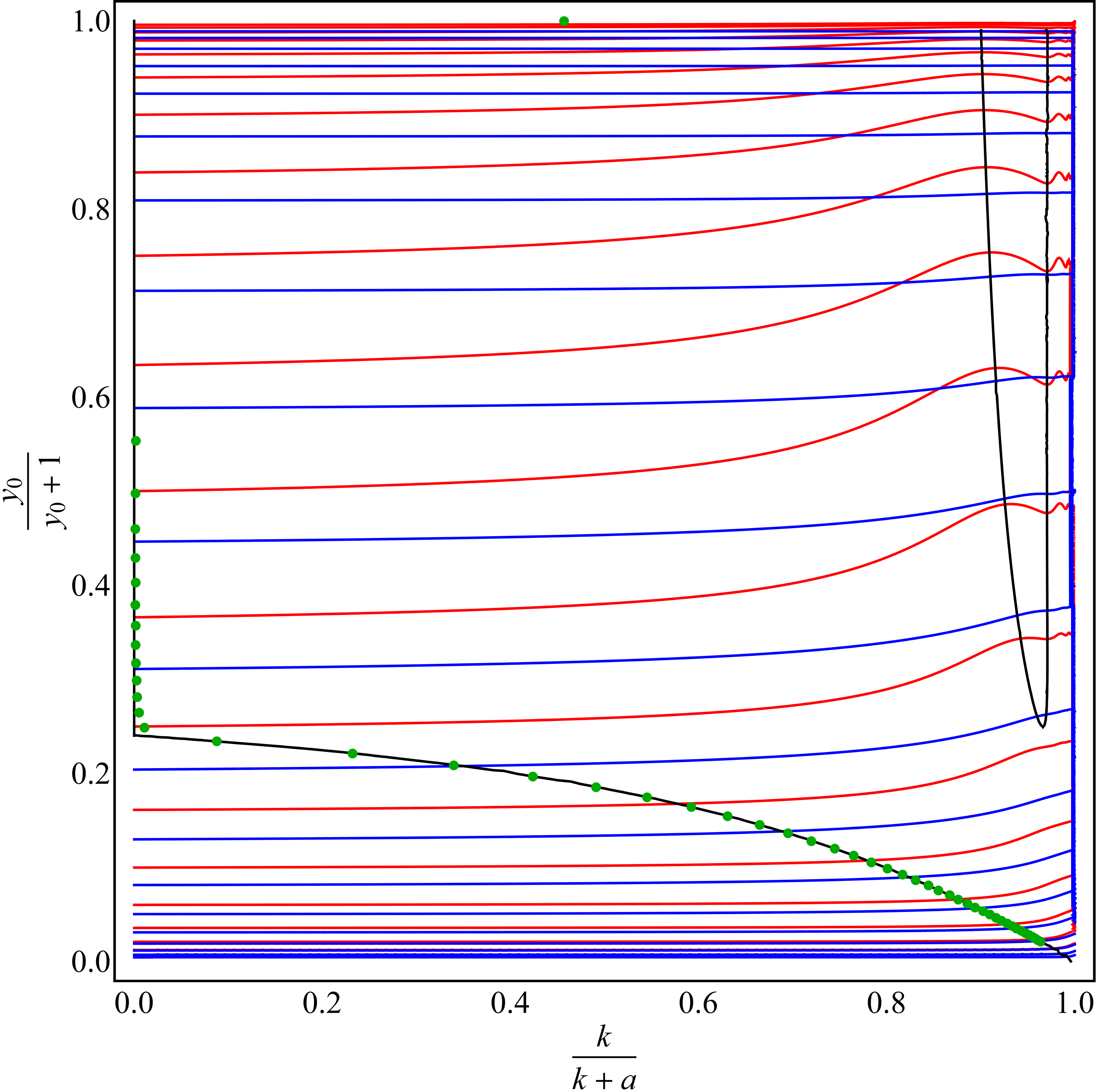

Supplement: Figure S3 — The Pareto front for a case of nonlinear integral feedback with no separation of time scales. We extend the model in the main text by adding a time dependent ODE for . In natural systems, the approximation that is much faster than is reasonable. We also added nonlinearity in which decay is multiplicative in , at rate . This is a reasonable model of damage repair systems in which the repair proteins interact by mass action kinetics with the damage . This results in . Performance contours are in red and blue. Black lines are lines where performance contours are externally tangent. Green dots are the Pareto front according to simulations (see Fig. 4s for details). The qualitative conclusions of the main text remain valid: Pareto front is a curve that connects the economy and efficiency archetypes. (TIF) [file pcbi.1003163.s003.tif]

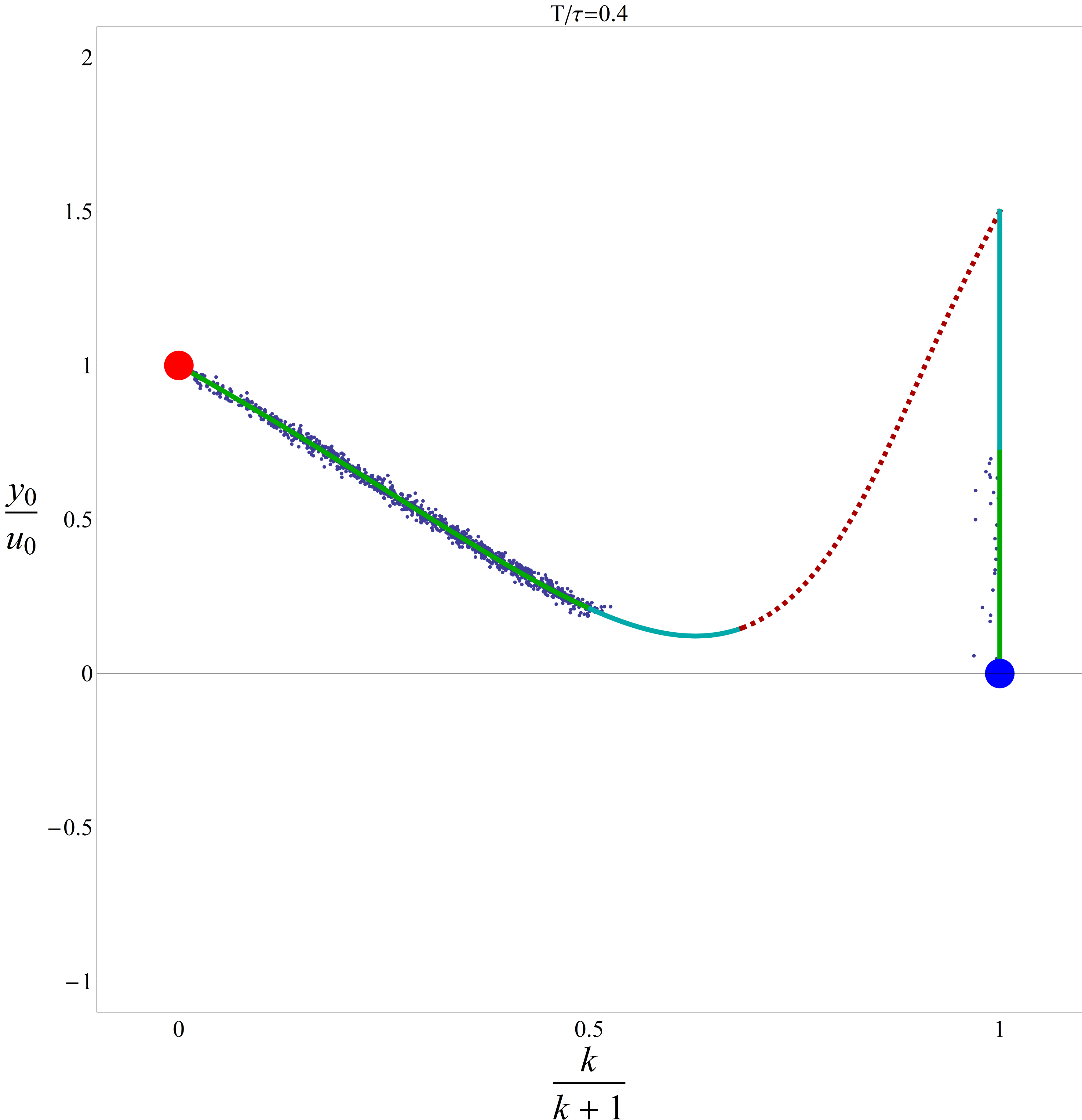

Supplement: Figure S4 — Simulations concur with the analytical results. Simulated data falls on the stable branches of the analytical solution for the Pareto front. Here, . Simulation used an initial population of randomly and uniformly distributed points. Points dominated in both tasks by other points were removed. Surviving points were perturbed by small noise (), and the process was repeated for 60 iterations, reducing the amplitude of the noise gradually to (). For comparison to Pareto simulation approaches see [75]–[78]. (TIF) [file pcbi.1003163.s004.tif]
